# Ergogenic Effects of Caffeine on Ballistic (Throwing) Performance: A Meta-Analytical Review

**DOI:** 10.3390/nu14194155

**Published:** 2022-10-06

**Authors:** Jozo Grgic, Dorian Varovic

**Affiliations:** 1 Institute for Health and Sport, Victoria University, Melbourne, VIC 3011, Australia; 2 Faculty of Kinesiology, University of Zagreb, 10000 Zagreb, Croatia

**Keywords:** dietary supplement, handball, shot put, javelin throw, athletics

## Abstract

Ballistic exercise is characterized by high velocity, force, and muscle activation. Typical examples of ballistic exercise are jumping and throwing activities. While several studies explored caffeine’s effects on throwing performance, the between study findings varied. Therefore, we performed a meta-analysis exploring caffeine’s effects on throwing performance (e.g., shot put, medicine ball throw, bench press throw). Seven databases were searched for eligible research. Ten studies (*n* = 151) were included. In the main meta-analysis, there was a significant ergogenic effect of caffeine on throwing performance (standardized mean difference [SMD]: 0.19; 95% confidence interval [CI]: 0.05, 0.33; *p* = 0.007). There was a significant ergogenic effect of caffeine in the subgroup analysis for studies that evaluated throwing velocity (SMD: 0.24; 95% CI: 0.10, 0.37; *p* = 0.0006) and used caffeine doses ≤3 mg/kg (SMD: 0.18; 95% CI: 0.05, 0.31; *p* = 0.006). There was no significant difference between caffeine and placebo in the subgroup analysis for studies that evaluated throwing distance (SMD: 0.15; 95% CI: −0.09, 0.40; *p* = 0.22) and used caffeine doses >3 mg/kg, (SMD: 0.17; 95% CI: −0.08, 0.41; *p* = 0.19). However, after one outlier study was excluded as part of a sensitivity analysis, an ergogenic effect was also observed for throwing distance and caffeine doses >3 mg/kg. Based on the results of this review, we conclude that individuals interested in the acute enhancement of throwing performance may consider caffeine supplementation.

## 1. Introduction

Ballistic actions are characterized by high motor unit firing rates, high rates of force development (RFD), and very brief contraction times [1]. Ballistic exercise differs from conventional resistance exercise [2]. In conventional resistance exercise, a large portion of the concentric phase involves deceleration followed by zero velocity at the end of the lift [3]. For example, in the bench press exercise performed with 80% of one-repetition maximum (1RM), around 50% of the concentric phase involves a deceleration phase [4]. However, ballistic exercises do not contain a deceleration phase, given that they require acceleration throughout the entire range of motion [2]. Additionally, in ballistic exercise, the external load or the athlete’s body is projected into a flight phase [2,3,4]. Due to these differences, ballistic exercises produce a higher velocity, force, and muscle activation when compared to traditional resistance exercises [2]. Typical examples of ballistic exercise are jumping and throwing activities [4].

Throwing performance is evaluated in several disciplines in athletics, such as the shot put, discus, hammer, and javelin throw [5]. In these disciplines, throwing distance directly influences placings. In addition to these sports, throwing performance is also important in sports such as baseball and handball [6]. Therefore, enhancing throwing performance may also impact sport-specific outcomes. In many sports, nutritional strategies are important in maximizing performance gains. Dietary guidelines have been provided for athletes competing in throwing events to enhance their performance [7]. For example, in 2019, Sygo et al. [7] provided a set of dietary and supplement recommendations. Among the outlined list of supplements, these authors suggested that athletes competing in throwing events should consider beta-alanine, creatine, nitrates, and caffeine. Caffeine is a highly popular ergogenic aid, with established performance-enhancing effects in various exercise modes [8,9,10]. However, an examination of the findings reported in primary studies highlights that caffeine’s effects on throwing performance are inconsistent [11,12,13,14,15,16,17,18,19,20]. Specifically, the first study [11] on this topic observed that caffeine consumption improved throwing performance in the shot put; however, an ergogenic effect was found only in one out of the six performed throwing attempts. Findings in subsequent studies varied, as some have also reported an ergogenic effect [14,15,16], while one study suggested that performance-enhancing benefits are attained only with higher caffeine doses [19]. At the same time, several studies [12,18] reported no significant difference between caffeine and placebo. Due to the conflicting data, specific recommendations regarding caffeine supplementation for throwing performance cannot be established.

As of date, there are no published reviews with a meta-analysis that pooled the data regarding caffeine’s effects on throwing performance. Such an analysis would be valuable given the conflicting results previously reported and the importance of throwing performance for various sports. Therefore, we aimed to perform a meta-analysis exploring caffeine’s effect on throwing performance.

## 2. Methods

### 2.1. Search Strategy

The primary search for studies that examined the effects of caffeine on throwing performance was conducted in seven databases: Academic Search Elite, SPORTDiscus, PubMed/MEDLINE, Scopus, Web of Science, Networked Digital Library of Theses and Dissertations, and Open Access Theses and Dissertations. To identify relevant studies, we applied the following combination of keywords and terms: caffeine AND (throw OR throwing OR “peak velocity” OR “medicine ball” OR “shot put” OR ballistic). In addition to searching the databases, we also performed secondary searches, which included: (a) screening the reference lists of the included studies (backward citation tracking); and (b) screening articles that referenced the included studies in Google Scholar (forward citation tracking). Finally, the personal collection of articles of the lead author was also examined. First, both authors of the review performed their independent searches. Upon completion, the lists of included studies were compared between authors and a consensus on the final included studies was made. The search process was performed on 26 September 2022.

### 2.2. Inclusion Criteria

To be included in the review, studies were required to satisfy the following PICO criteria:

- P (population)—apparently healthy adults;
- I (intervention)—caffeine consumption provided before exercise;
- C (comparison)—placebo condition;
- O (outcomes)—throwing distance (e.g., shot put distance, medicine ball throwing distance) or throwing velocity (e.g., velocity in the bench press throw).

In addition to the PICO criteria, studies were also required to be published in English and as a full-text manuscript (conference abstracts were not considered).

### 2.3. Data Extraction

From all the included studies, the following data were extracted:

- Last name from the lead author and year of manuscript publication;
- Study design;
- Participants’ characteristics (e.g., sample size, training status, sex);
- Caffeine supplementation protocol, including caffeine dose, caffeine source, and timing of consumption;
- Throwing performance test and its outcome.

Finally, performance outcome mean ± standard deviation data from the caffeine and placebo were extracted for statistical analysis. Data extraction was performed independently by the two authors of the review to minimize possible errors.

### 2.4. Risk of Bias Assessment

Risk of bias was evaluated using the RoB 2 tool, which also contained considerations for crossover trials [21]. Risk of bias is evaluated based on six domains, including bias that may arise: from the randomization process (domain 1); from period and carryover effects (domain S); due to deviations from intended intervention (domain 2); because of missing data (domain 3); due to the measurement of the outcome (domain 4); due to selection of the reported result (domain 5). Individual domains and the overall risk of bias are classified as “low risk”, “some concerns”, or “high risk” [21].

### 2.5. Statistical Analysis

A random-effects meta-analysis of standardized mean differences (SMD) was performed to compare the effects of caffeine vs. placebo on throwing performance. SMDs were calculated as the difference in the means between the caffeine and placebo trials divided by their pooled standard deviation. In addition to the mean and standard deviation values, the sample size and correlation between trials are also required in the calculation of SMD. However, the included studies did not report correlation values. Nevertheless, we had access to correlation values from two of the included studies [15,19]. Correlation in these two studies ranged from 0.84 to 0.94. The average value of *r* = 0.89 was used for all studies in the analysis. Several studies provided multiple related performance outcomes (e.g., throwing performance following the ingestion of two different caffeine doses) [11,13,14,17,18,19,20]. As these were outcomes collected in the same group of participants, SMDs and variances were first calculated separately for each outcome. Then, the average SMD and variance values were used in the meta-analysis. In the main meta-analysis, all the included studies were analyzed. Subgroup analyses were performed according to throwing performance outcomes and caffeine doses. Specifically, subgroup analyses were performed to examine caffeine’s effects on throwing distance and throwing velocity. Subgroup analyses also examined the effects of caffeine in doses ≤3 mg/kg vs. >3 mg/kg. For each of these outcomes, sensitivity analyses were performed by excluding the data from one study [12], which was classified as an outlier based on the visual inspection of the forest plot. SMDs were interpreted as trivial (<0.20), small (0.20–0.49), medium (0.50–0.79), and large (≥0.80) [22]. Heterogeneity was examined using *I^2^* and interpreted as low (≤50%), moderate (50–75%), and high (>75%) levels of heterogeneity. The statistical significance was tested at *p* < 0.05. The Comprehensive Meta-analysis software version 2 (Biostat Inc., Englewood, NJ, USA) was used for data analysis. 

## 3. Results

### 3.1. Search Results

**Figure 1:**
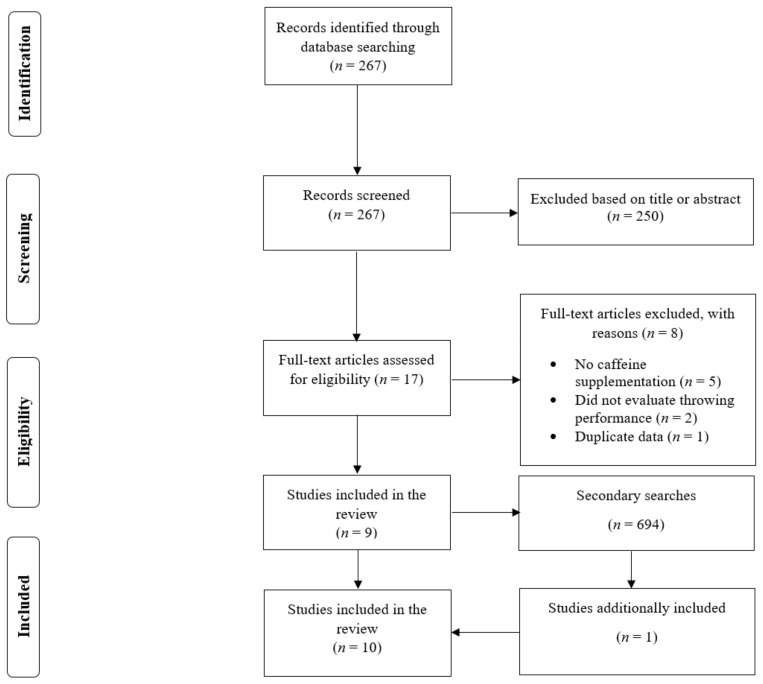
Flow diagram of the search process.

When examining the databases, there were 267 results. Out of the 267 references, 250 articles were excluded because they were duplicates or they were deemed not relevant for this review after reading their titles or abstracts. Thus, 17 full-text articles were read. Nine studies were excluded, most commonly because they did not provide isolated caffeine supplementation or did not evaluate ballistic throwing performance [23,24,25,26,27,28,29,30,31]. As a result, nine articles [11,12,13,14,15,17,18,19,20] were found to satisfy the inclusion criteria in the primary search. There were 694 search results in the secondary search, even though no additional studies were found to satisfy the inclusion criteria. One study [16] from the lead author’s personal collection of articles was found to satisfy the inclusion criteria. Therefore, ten studies were included in the review [11,12,13,14,15,16,17,18,19,20] (Figure 1). While there were ten included studies, there were 11 groups included in the meta-analysis due to the fact that one study [17] had two groups of participants who possessed different genotype variations. These groups were independent participants with their individual data recorded for the caffeine and placebo trials and were therefore analyzed separately.

### 3.2. Summary of the Included Studies

All the included studies used either a double-blind or triple-blind design. Studies were published between 2012 and 2022 (Table 1). The median number of participants per study was 13, while the combined number of participants was 151 (40 females and 111 males). Six studies included resistance-trained participants, two studies included handball players, and two studies included shot putters. Eight studies provided caffeine in liquid or capsule form in doses relative to body mass, ranging from 2 to 12 mg/kg. Two studies provided either caffeine gum (100 mg of caffeine) or caffeine gel (75 mg of caffeine). Four studies evaluated performance using medicine ball throw tests (2, 5, or 9 kg medicine ball weight). Three studies evaluated bench press throw performance using only the barbell or an external load of 30% of 1RM. Two studies evaluated shot put performance using weights of 4 kg for females and 7.26 kg for males.

### 3.3. Risk of Bias

For domains 1 and 5, all included studies were classified as having “some concerns” regarding the risk of bias. In all other domains, the evaluation was recorded as “low risk” of bias. The overall evaluation of the risk of bias for all included studies was recorded as “some concerns”.

### 3.4. Meta-Analysis Results

**Figure 2:**
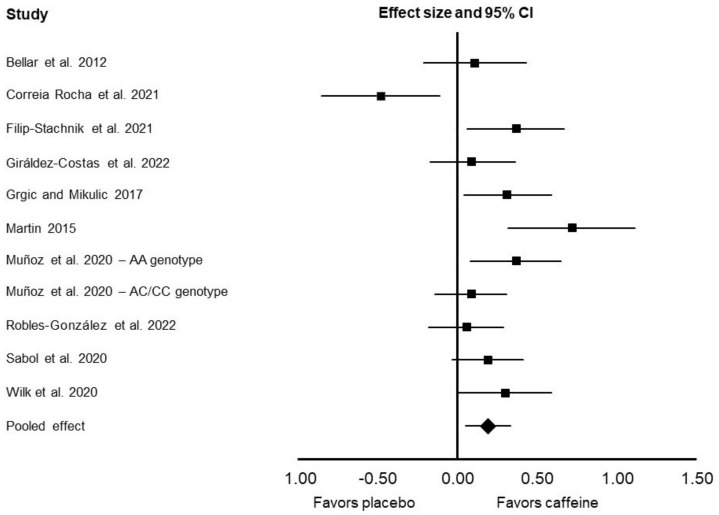
The forest plot presents the results of the random-effects meta-analysis that compared the effects of caffeine ingestion vs. placebo on throwing performance. The results are presented as standardized mean differences (effect size) and their 95% confidence intervals (CI). The squares present effect sizes and the whiskers are 95% CI. The diamond-shaped effect size represents the overall (pooled) effect, which indicates favoring of caffeine when compared to placebo for throwing performance. The meta-analysis was performed using data from ten included studies [11,12,13,14,15,16,17,18,19,20].

In the main meta-analysis, there was a significant ergogenic effect of caffeine on throwing performance (SMD: 0.19; 95% confidence interval [CI]: 0.05, 0.33; *p* = 0.007; *I*^2^ = 62%; Figure 2). In the sensitivity analysis, the ergogenic effect of caffeine slightly increased (SMD: 0.23; 95% CI: 0.12, 0.33; *p* < 0.0001; *I*^2^ = 32%).

In the subgroup analysis for studies that evaluated throwing distance, there was no significant difference between caffeine and placebo (SMD: 0.15; 95% CI: −0.09, 0.40; *p* = 0.22; *I*^2^ = 76%). In the sensitivity analysis, there was an ergogenic effect of caffeine on throwing distance (SMD: 0.25; 95% CI: 0.07, 0.43; *p* = 0.007; *I*^2^ = 51%). In the subgroup analysis for studies that evaluated throwing velocity, there was a significant ergogenic effect of caffeine (SMD: 0.24; 95% CI: 0.10, 0.37; *p* = 0.0006; *I*^2^ = 28%).

In the subgroup analysis for studies that used caffeine doses ≤3 mg/kg, there was a significant ergogenic effect of caffeine on throwing performance (SMD: 0.18; 95% CI: 0.05, 0.31; *p* = 0.006; *I*^2^ = 41%). In the subgroup analysis for studies that used caffeine doses >3 mg/kg, there was no significant difference between caffeine and placebo for throwing performance (SMD: 0.17; 95% CI: −0.08, 0.41; *p* = 0.19; *I*^2^ = 73%). In the sensitivity analysis, there was an ergogenic effect of caffeine doses >3 mg/kg on throwing performance (SMD: 0.29; 95% CI: 0.16, 0.43; *p* < 0.0001; *I*^2^ = 0%).

## 4. Discussion

Our main finding is that caffeine ingestion enhances throwing performance. In subgroup meta-analyses, ergogenic effects of caffeine were found for outcomes related to throwing velocity and doses ≤3 mg/kg. There was no significant difference between caffeine and placebo for throwing distance outcomes and caffeine doses >3 mg/kg. However, when an outlier study was excluded as a part of a sensitivity analysis, ergogenic effects in these subgroups were also found. In summary, individuals interested in the acute enhancement of throwing performance may consider caffeine supplementation.

Based on the results of this review, caffeine ingestion is ergogenic for throwing performance. Previous studies have established that ballistic exercise is characterized by high RFD [32]. This is important to consider, given the recent findings also supporting an ergogenic effect of caffeine on RFD [33]. One of the determinants of RFD is motor unit recruitment [33]. This is relevant to mention as motor unit recruitment may be increased following caffeine ingestion [34]. Due to these effects, it seems likely that caffeine ingestion increases motor unit recruitment, resulting in an increase in RFD and enhancement of throwing performance [32,33,34].

While there are no previous meta-analytical data regarding caffeine’s effects on throwing performance, such data exists for other ballistic exercise forms. One meta-analysis explored the effects of caffeine on jump height [35]. Ten studies were included, and a small ergogenic effect of caffeine was found (SMD: 0.17). Another meta-analysis explored the effects of caffeine on jump height in single and repeated jumps performed by team sport athletes [36]. As with the initial findings, ergogenic effects in the SMD range from 0.19 to 0.29 were found [36]. Finally, recent meta-analytical data also established an ergogenic effect of low (<3 mg/kg) caffeine doses on jump height (SMD: 0.21) [37]. Overall, the magnitude of caffeine’s effects on jumping performance is nearly identical to those observed herein (SMD: 0.18 to 0.29). Therefore, our results add to the evidence base by establishing a similar ergogenic effect of caffeine for throwing performance, in which upper-body musculature was predominantly involved. While such improvements in performance may seem small, it should be mentioned that changes as small as 0.9 to 1.5% represent a worthwhile improvement in the performance of throwing events in athletics [38].

Current caffeine supplementation recommendations are to use doses ranging from 3 to 6 mg/kg for an ergogenic effect [39]. One of the included studies used a dose–response design with caffeine in doses amounting to 2, 4, and 6 mg/kg [19]. Compared to the placebo condition, the SMD increased from 0.07, 0.24, to 0.27 for caffeine doses of 2, 4, and 6 mg/kg, respectively. Statistically significant differences were only observed between placebo and 6 mg/kg of caffeine. These results suggested that higher caffeine doses may be needed to enhance throwing performance. However, in the subgroup analysis for studies that used caffeine doses ≤3 mg/kg, caffeine still had a significant ergogenic effect (SMD: 0.18). As such, these results agree with a recent meta-analysis reporting that caffeine doses <3 mg/kg are ergogenic for other forms of ballistic exercise (i.e., jumping) [37]. A limitation of the subgroup analysis for caffeine doses ≤3 mg/kg is that two of the included studies [11,16] provided absolute doses (i.e., 100 and 75 mg). Such an approach certainly has limitations, given that the relative dose will inherently vary among the included participants, which may contribute to variations in individual responses. While an ergogenic effect was observed with smaller doses, there were no significant differences between caffeine and placebo for caffeine doses >3 mg/kg. However, the lack of significant improvements was largely influenced by one outlier study [12]. After this study was excluded as a part of a sensitivity analysis, an ergogenic effect of doses higher than 3 mg/kg was also observed (SMD: 0.29). Overall, smaller caffeine doses also provide an ergogenic effect on throwing performance, even though future dose–response studies are needed, especially with caffeine provided in relative doses.

As previously mentioned, one study [12] was identified as an outliner through the visual inspection of the forest plot. Specifically, the performance data in this study highly favored the placebo condition. This contrasts with all other studies in which the data were towards the “favors caffeine” side of the forest plot (Figure 2). The reasons for these discrepancies in the direction of effects are not fully clear, given that the referenced study used a similar supplementation protocol as all the other studies. Still, the researchers used a lower-weight medicine ball (2 kg vs. 5 or 9 kg) compared to other studies. There might be a certain threshold of external load that needs to be used to observe an ergogenic effect of caffeine. Additionally, the differences in findings might be due to the substantial inter-individual variability in responses between the participants. Inspection of the individual participant data highlighted that some participants performed substantially better after placebo ingestion. For example, one participant recorded a 20% performance improvement following placebo ingestion. The authors, however, did not report on some important information, such as the participants’ habitual caffeine intake and at which time of day the testing was performed [40,41]. It might be that some of these variables contributed to the variability in responses, and future studies should ensure that these data are reported to allow for a better extrapolation of their findings.

There are several limitations of this review that need to be considered when interpreting the results. While the included studies were double- or triple-blinded, there were still classified as having “some concerns” on the RoB 2 checklist. Studies were classified as having “some concerns” as they did not report on allocation concealment and were not pre-registered. Additionally, out of the 151 included participants, 74% were males. Therefore, the predominance of collected evidence is in the male population. Several studies included both sexes, but did not examine sex-specific effects [11,14,17]. One study [18] included only females and reported that caffeine ingestion did not enhance throwing performance. Even though recent studies [42,43,44] suggest that males and females respond similarly to caffeine, future studies on the female population are nonetheless needed. While this is not necessarily a limitation of the present review, future studies are also needed to explore the effects of caffeine among participants with varying genotypes and different caffeine sources (e.g., gels, capsules, coffee) [45,46,47]. Such findings would aid in refining and individualizing the guidelines for caffeine supplementation.

## 5. Conclusions

The present meta-analysis examined caffeine’s effects on throwing performance. The meta-analytical data presented herein found that caffeine ingestion enhances throwing performance. The magnitude of caffeine’s ergogenic effect may be classified as small but may be of relevance in sporting tasks that involve ballistic throwing. Caffeine doses ≤3 mg/kg or >3 mg/kg are likely to be ergogenic for throwing distance and throwing velocity. Future studies on the female population are required to increase the generalizability of these findings.

## Figures and Tables

**Table 1:** Summary of the included studies.

Study	Participants	Caffeine Protocol	Throwing Test
Bellar et al. 2012 [11]	9 college shot putters (4 males/5 female)	100 mg in gum, 15 min before exercise	SP (4 kg for females, 7.26 kg for males)
Correia Rocha et al. 2021 [12]	10 male handball players	5 mg/kg in capsules, 50 min before exercise	MBT (2 kg)
Filip-Stachnik et al. 2021 [13]	12 resistance-trained males	9 or 12 mg/kg in capsules, 60 min before exercise	BPT with 30% of 1RM
Giráldez-Costas et al. 2022 [14]	13 shot putters (8 males/5 females)	3 mg/kg in capsules, 45 min before exercise	BT, SSP, and SP (4 kg for females, 7.26 kg for males); BPU
Grgic and Mikulic 2017 [15]	17 resistance-trained males	6 mg/kg in liquid, 60 min before exercise	MBT (9 kg)
Martin 2015 [16]	12 resistance-trained males	75 mg in gel, 60 min before exercise	MBT (5 kg)
Muñoz et al. 2020 [17]	31 professional handball players (16 males/15 females)	3 mg/kg in capsules, 60 min before exercise	7-m and 9-m distance HT
Robles-González et al. 2022 [18]	15 resistance-trained females	3 mg/kg in liquid 30 min, before exercise	BPT using only the barbell (17 kg)
Sabol et al. 2020 [19]	20 resistance-trained males	2, 4, or 6 mg/kg in capsules, 60 min before exercise	MBT (9 kg)
Wilk et al. 2020 [20]	12 resistance-trained males	3 or 6 mg/kg in capsules, 60 min before exercise	BPT with 30% of 1RM

1RM: one-repetition maximum; SP: shot put; MBT: medicine ball throw; BPT: bench press throw; HT: handball throw; BT: backwards throw; SSP: standing shot put; SP: shot put; and BPU: ballistic push-up.

## Data Availability

Data used for the analysis are available from the corresponding authors on request.
